# Approaches for the Identification of Genetic Modifiers of Nutrient Dependent Phenotypes: Examples from Folate

**DOI:** 10.3389/fnut.2014.00008

**Published:** 2014-07-14

**Authors:** John W. R. Zinck, Amanda J. MacFarlane

**Affiliations:** ^1^Science Integration Division, Public Health Agency of Canada, Ottawa, ON, Canada; ^2^Nutrition Research Division, Health Canada, Ottawa, ON, Canada

**Keywords:** folate metabolism, folate, folic acid, functional genomics, candidate gene, genome-wide association studies

## Abstract

By combining the sciences of nutrition, bioinformatics, genomics, population genetics, and epidemiology, nutrigenomics is improving our understanding of how diet and nutrient intake can interact with or modify gene expression and disease risk. In this review, we explore various approaches to examine gene–nutrient interactions and the modifying role of nutrient consumption, as they relate to nutrient status and disease risk in human populations. Two common approaches include the use of SNPs in candidate genes to identify their association with nutritional status or disease outcomes, or genome-wide association studies to identify genetic polymorphisms associated with a given phenotype. Here, we examine the results of various gene–nutrient interaction studies, the association of genetic polymorphisms with disease expression, and the identification of nutritional factors that modify gene-dependent disease phenotypes. We have focused on specific examples from investigations of the interactions of folate, B-vitamin consumption, and polymorphisms in the genes of B-vitamin dependent enzymes and their association with disease risk, followed by an examination of the strengths and limitations of the methods employed. We also present suggestions for future studies, including an approach from an on-going large scale study, to examine the interaction of nutrient intake and genotypic variation and their impact on nutritional status.

## Application of Genomics to Address Public Health

Specific sub-populations may be genetically pre-disposed to high or low status for specific nutrients. New high-throughput sequencing and bioinformatics tools for genetic analysis have opened the door to performing detailed analyses of the interrelationships among dietary intake, nutritional status, and genetic polymorphisms. Data from these studies may in turn be used to inform nutrition policies to maximize public health benefits. For example, we are currently identifying single nucleotide polymorphisms (SNPs) that predict an individual’s response to folic acid intake in the Canadian population using samples from the Canadian Health Measures Survey cycle 1 (1). In this review, we explore various approaches to examine gene–nutrient interactions and the effect of nutrient consumption on gene expression and function as they relate to disease risk in human populations. We present specific examples from investigations of the interactions of folate and B-vitamin consumption and polymorphisms in the genes of B-vitamin dependent enzymes and their association with disease risk.

## Gene–Environment Interaction

Since the advent of DNA sequencing, numerous genetic polymorphisms have been identified that are associated with human diseases including developmental anomalies, diabetes, cardiovascular disease, and cancer (2, 3). SNPs can play an integral role in phenotypic plasticity and contribute to much of the genetic variability observed in phenotypes dependent on gene–environment interactions (4). Although SNPs have been associated with chronic diseases or disorders, in general, a single SNP or group of SNPs (haplotype) is rarely by itself causal (5). Put simply, SNPs are often necessary, but not sufficient, for the development of a given disease.

**Figure 1:**
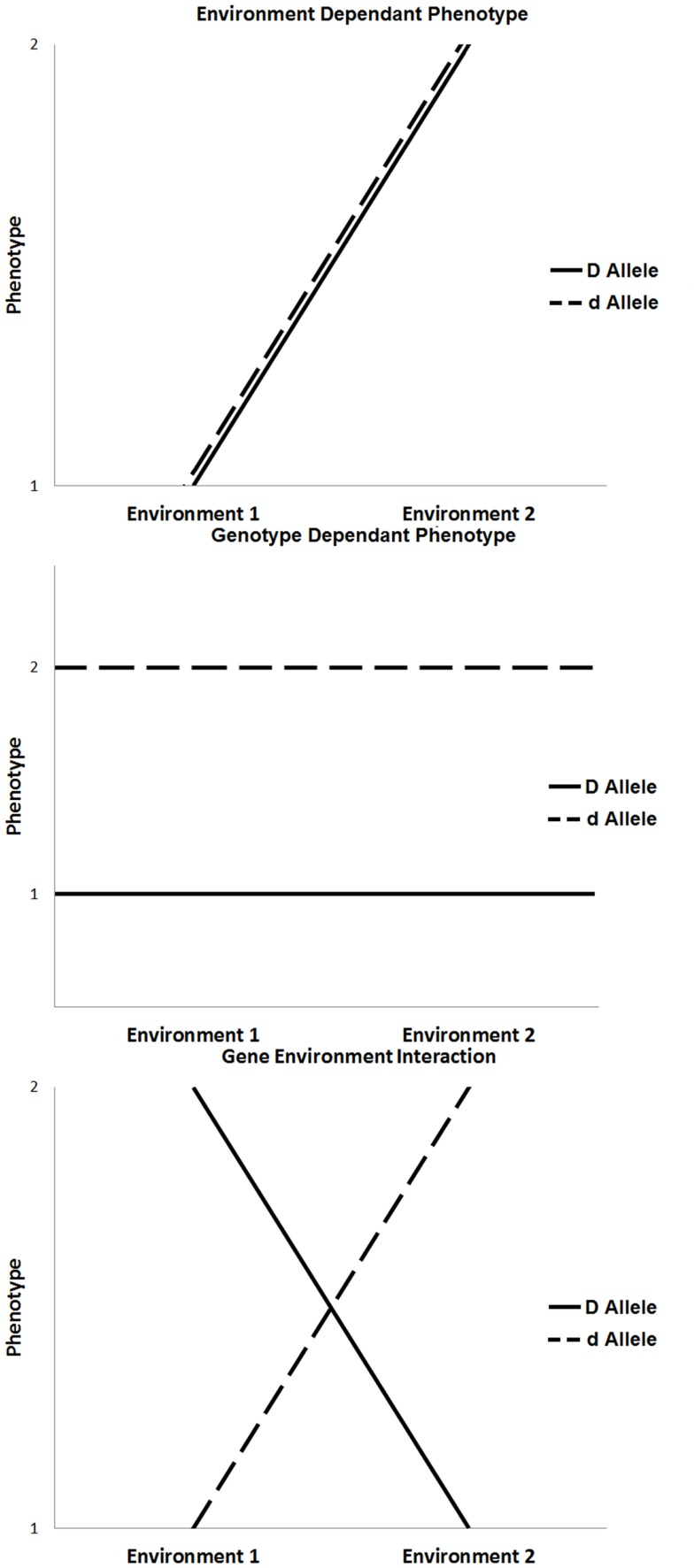
**Three scenarios that influence phenotypic expression, such as environment only influence, genotype only influence, and the influence of gene × environment interaction**.

The majority of chronic diseases are multifactorial and depend on gene–environment interactions [(5, 6); Figure 1]. The influence of environmental factors alters the phenotypic expression of a genotype. In the context of disease, these interactions may result in increased susceptibility or resistance depending on both the genotype and environmental exposure. Environmental factors include any non-genetic contribution to a phenotype, which could directly influence the output of a genetic pathway to prevent, initiate, aggravate, or mitigate a pathological response. These can be under an individual’s control, such as behavioral or nutritional factors, or they may be beyond an individual’s control, such as climate change or unintended exposure to toxins (7).

The outcome of gene–environment interactions can cause a change in genotype ranking (genotypic advantage changes in different environments), a change in scale of phenotypic expression, or a combination of both. Various experimental models of gene–environmental interaction have been developed to account for ways in which genetic effects can be modified by environmental exposures, taking into account the number of exposures, the level of exposure, the timing of exposure, and the model on which the genetic effects are based (8, 9). For example, the most basic gene–environment interactions include a single environmental factor that alters the expression of a single gene to influence phenotype. In the absence of one or the other, the disease will not occur. More commonly, the expression of a disease phenotype, such as diabetes or cardiovascular disease, is dependent on multiple genes (gene–gene interactions), and single or multiple environmental exposures. In these more complex conditions, any given individual gene or environmental factor is a minor contributor to disease expression; rather, it is the combined effect of multiple genes modified by specific environmental triggers that, when combined, result in disease.

**Figure 2:**
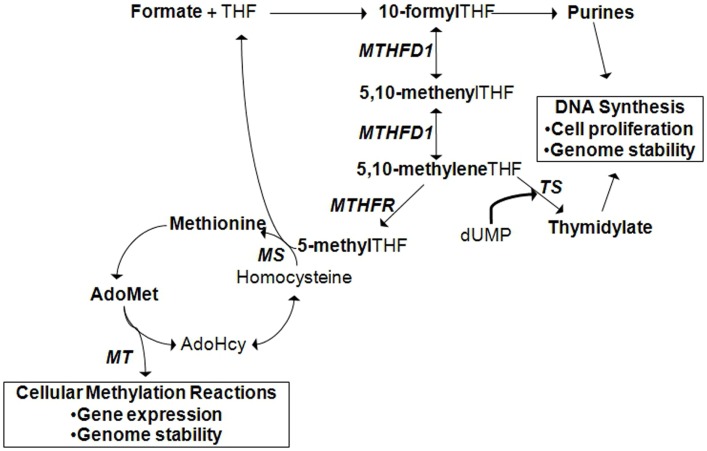
**Cytoplasmic folate-mediated one-carbon metabolism is required for *de novo* purine, thymidylate, and methionine synthesis**. Cellular methylation potential is dependent on the production of *S*-adenoyslmethionine (AdoMet), the major methyl donor in the cell. Methionine is generated when homocysteine is remethylated. A methyl group can be transferred from 5-methyltetrahydrofolate (THF) by methionine synthase (MS). 5-methylTHF is produced by methylene THF reductase (MTHFR) from the folate metabolic pathway. In the liver and kidney, homocysteine can also be remethylated by betaine:homocysteine methyltransferase (BHMT), a reaction that depends on choline-derived betaine as the methyl donor (not shown). Methionine is converted to AdoMet, from which methyltransferases (MT) transfer methyl groups to acceptor molecules. Acceptor molecules include DNA, RNA, histones and other proteins, and other small molecules. The reaction produces *S*-adenosylhomocysteine (AdoHcy) that can be converted to homocysteine. TS, thymidylate synthase; MTHFD1, methylene THF dehydrogenase 1.

It must be mentioned that one mechanism by which environmental factors can influence gene expression is through epigenetics. Epigenetics is defined as a change in gene expression in the absence of a change to the DNA sequence (10). Epigenetic regulation of gene expression can occur by DNA methylation, methylation or other post-translational modifications of histones, or the activation of microRNAs. In addition, recent studies have revealed that epigenetic variation can interact with genetic variation in the expression of a disease phenotype (11). The concept of epigenetic gene regulation is pertinent in the context of folate metabolism since folate status influences cellular methylation capacity (Figure 2; see also section: *Case in point: Nutrigenomic approaches in the study of folate-related pathologies*). However, for succinctness, we will limit the scope of our discussion to the interaction between environmental factors and genetic variants.

## Nutrient Dependant Genetic Associations

Nutrients represent a large, varied, and ubiquitous group of environmental factors involved in gene–environment interactions. The study of gene–nutrient interactions has begun to identify the underlying roles of specific nutrients in disease and developmental disorders. By understanding the mechanisms underlying gene–nutrient interactions, the nutrition of both individuals and populations may be tailored to reduce the burden of chronic disease. These studies are often based on observed associations between the intake and status of a nutrient with risk for a disease. These associations are often initially observed in case–control, cohort, or longitudinal studies, as well as in animal models. Though, these studies are unable to pinpoint the underlying genetic factors contributing to phenotype expression, they link specific nutrients to a particular disease and provide the foundation on which hypothesis driven experiments can be developed. For example, these types of studies have demonstrated an association between higher serum levels of Vitamin A and reduced risk of lung cancer (12), nutrition in early development and later cognitive development (13), and low maternal folate status and increased neural tube defect (NTD) risk (14).

Two approaches to study how nutrients interact with genes to influence phenotype have been widely applied. The first is the candidate gene approach, which examines the relationship between SNPs in candidate genes (usually nutrient-related genes) and disease risk. The second, GWAS, surveys genetic polymorphisms in a genome-wide fashion to assess their association with disease risk (15, 16). Finally, the modifying effect of nutrient intake on the associations between genetic polymorphisms and disease phenotype, or its effect on gene expression in disease can also be examined. With the increasing speed and accuracy of whole genome sequencing, the ability to identify, and characterize these interactions has increased dramatically.

## Functional Genomics for Nutrigenomics

With the development of high-throughput, genomic sequencing technologies, it is feasible to examine links among nutrition, genetics, and phenotype. The majority of nutrigenomic studies have used either a candidate gene or genome-wide association study (GWAS) approach to examine the interrelationship among SNPs or other genetic polymorphisms, nutrient intake, nutrient status, and disease (Table 1).

**Table 1:** **A comparison of two major methods used to conduct gene association studies, candidate gene studies, and genome-wide association studies**.

**GENIC ASSOCIATION APPROACH**	
**Candidate gene studies**	
Methodology	Examine associations between genetic polymorphisms and environmental interactions within pre-specified genes of interest
	Hypothesis driven, case–control for binary outcomes or single cohort for continuous associations
	Requires *a priori* knowledge of functional relationship between genes and target traits
	Increasingly, relationship information is available in online databases
Limitations	Reliance on existing information may limit scope of examined causative genes
	Initial costs associated with identifying target genes and their function can be high if not previously published
**Genome-wide association studies (GWAS)**	
Methodology	Examines many (tens or hundreds of thousands) of genetic variants
	Contrast two large groups of individuals using case vs. control or a continuous outcome to determine if allelic patterns are significantly associated with a trait of interest
	Requires no prior knowledge of relationship between gene function and phenotypic traits
Limitations	Requires rigorous quality control to limit false-positive results caused by multiple pairwise comparisons
	Large dataset size, lack of functional model testing and confounding biological and environmental factors that are not, or cannot be considered can lead to erroneous associations
	Common genetic variants used for some GWAS may not play a role in explaining the heritable variation of common disease

Candidate gene association studies are preferred when the functional relationship of the target genes with an outcome is known (17, 18). The reliance on sometimes limited *a priori* information and the inability to consider all causative genes for a given trait may hinder the scope of candidate gene studies (18–20). New sequencing techniques with faster analysis times are addressing this criticism, in part, by providing a means to economically examine many genetic variants within a candidate gene and expanded sets of candidate genes quickly. Unlike candidate gene studies, GWAS require no prior knowledge of a gene’s functional relationship to a given nutrient. In a GWAS, a suite of genetic markers, commonly SNPs, is screened to determine if the frequency of allele variants is significantly different between the case and control groups (21). Of the many advantages of GWAS, the ability to compare SNP allele frequency with quantitative environmental and phenotypic data may be the most important.

Finding which loci in a suite of 100,000 (or more) markers have a significant association with a phenotype presents a difficult statistical problem, similar to finding a needle in a haystack. The statistical power of a GWAS is a function of sample size, effect size, causal allele frequency, and marker allele frequency and its correlation with causal variants. When examining multiple associations, GWASs can be fundamentally underpowered when the association is of modest effect (22). Although the action of multiple factors (genetic or non-genetic), incomplete penetrance, and modest effects reduce analytical power, proper study design, with subjectively selected (large) population sizes, can overcome these limitations (23). However, even in cases with adequate sample size, GWAS have a high potential for false-positive results because of the massive number of markers and statistical tests applied (24). The risk of false positives can be avoided through proper quality control and study design, including using appropriate analytical methods to correct for multiple testing [e.g., Bonferroni corrected *p*-value, false discovery rate, permutation testing, BitForce (25); network-assisted investigation (26); high-dimensional vectors (27)].

The GWAS approach has evolved with new sequencing and computational technology, and now includes random SNP array selections, SNP functional predictions, and exomic SNP analyses that theoretically cover only coding regions of the genome. In these approaches, tag SNPs, a single-marker in a region of high linkage-disequilibrium that can be used to represent the allele frequencies of a group of SNPs, are popular as they dramatically reduce the time and costs associated with examining suites of genetic markers without losing resolution (28).

## Case in Point: Nutrigenomic Approaches in the Study of Folate-Related Pathologies

Folate is an essential water-soluble B-vitamin that is required for metabolic one-carbon transfers in the *de novo* synthesis of purines, thymidylate, and methionine [Figure 2; (29)]. As such, adequate folate consumption is essential for the synthesis, stability, and repair of DNA (29). In conditions of folate deficiency, uracil can be incorporated into DNA in the absence of thymidylate, which can lead to DNA strand breakage and reduced DNA repair. Reduced purine synthesis results in reduced cell proliferation, cell stasis, and death. Folate deficiency also results in increased homocysteine production, a one-carbon metabolism by-product related to cardiovascular disease and other pathologies, due to reduced methionine synthesis (30–32). Reduced methionine synthesis limits the availability of *S*-adenosylmethionine for cellular methylation reactions. Folate deficiency, resulting from inadequate intake or genetic polymorphisms in folate-dependent genes, is associated with increased risk for NTDs, cancer, cardiovascular diseases, and other chronic diseases.

Folate-dependent one-carbon metabolism and homeostasis requires a number of transporters, enzymes, and other nutrient co-factors. Nearly all folate-related genetic studies have used candidate gene or GWAS approaches to identify genetic markers associated with a folate-dependent disease or for the identification of associations among nutrient intake, folate-related genes, and functional outcomes. Screening of more than 1500 SNPs identified in folate and other B-vitamin metabolic pathways has revealed ~150 SNPs with significant associations with pathological endpoints (33, 34). Commonly, studies have either examined the relationship between one or a few genes and nutrient status and/or disease. Others have performed large GWAS analyses to identify the association of genetic polymorphisms with a single phenotype (e.g., disease or nutritional status), but they do not consider confounding variants. The limitations of these studies include limited population size, limited number of candidate genes (1–10, e.g., *MTHFR*, Methylenetetrahydrofolate reductase, studies; Table 2) and/or lack of dietary/supplement intake data or status data. Here, we will discuss three diseases for which relationships with folate and folate-dependent genes have been studied: NTDs; intestinal cancers; and, cardiovascular disease.

**Table 2:** **Major findings from association studies between *MTHFR* gene variants and two proof of principle diseases, neural tube defects, and cancer risk, including sample size, significance (as *p* or 95% confidence interval), and odds ratio (OR)**.

Research Study	Approach	Findings	Sample Size	Significance	OR
***MTHFR* AND NTDs**					
Boyles et al. (35)	GWAS, clinical sample population	No significant *MTHFR* SNP (rs1801133)/NTD risk associations in individuals not taking maternal folate supplements	Total: 304	*p*: 0.203	
Shaw et al. (33)	Candidate gene, large sample population	Significant association between *MTHFR* SNP (rs1801133) and NTD risk. No link between intake and allele frequency	Cases: 259, controls: 359	95% CI: 1.2–3.1	2.0
Pangilinan et al. (34)	tagSNP	Significant association between *MTHFR* SNPs (rs17037425, rs17367504) and NTD risk	Cases: 301, controls: 341	*p*: 0.0068, *p*: 0.0081	
***MTHFR* AND CANCER RISK**					
Vollset et al. (36)	GWAS	No correlations between gastric cancer and plasma folate, total homocysteine and serum B12. Slight increase in gastric cancer risk with *MTHFR* (rs1801133).	Cases: 245, controls: 631	*p*: 0.04	1.47
Ibiebele et al. (37)	Candidate gene	No link between *MTHFR* (rs1801131, rs1801133) and esophageal adenocarcinomas.	Cases: 881, controls: 1507	*p*: 0.22, *p*: 0.72	
Terry et al. (38)	Candidate gene	*MTHFR* SNPs (rs1801131, rs1801133) showed no significant association with increased ovarian cancer risk.	Cases: 1642, controls: 2068	*p*: 0.59, *p*: 0.58	
Han et al. (39)	GWAS	Link between *MTHFR* (rs1801131) and colorectal adenoma in individuals with extremely low folate intake.	Case: 1331, controls: 1501	*p*: 0.02	

## Neural Tube Defects

Folate consumption and status during the peri-conceptional period has long been associated with a reduction in developmental anomalies, including NTDs (14, 40–42). During pregnancy, NTDs occur between 21 and 28 days after conception if the neural plate fails to fold properly to form the neural tube, the precursor of the spinal cord and brain. When the neural tube fails to close, numerous NTD defects can arise including spina bifida and anencephaly (43). Adequate maternal peri-conceptional folic acid supplementation significantly reduces the risk of primary or recurrent NTDs (14, 40). In an effort to reduce the number of NTD-affected pregnancies, many countries, including Canada and the United States, have mandated folic acid fortification of white flour and other cereal products (44–46). In Canada, NTD incidence has decreased by ~45% since the start of fortification (47). The specific mechanism(s) by which folate prevents NTDs remains poorly understood, but its role in nucleotide synthesis, DNA repair and cellular methylation reactions are likely important.

The early observation of a high recurrence rate of NTDs pointed to possible genetic contributors to pathogenesis. Numerous studies have sought to identify the underlying genetic factors influencing NTD prevalence (48–52). The genetic link combined with the evident association between maternal folate status and NTDs led to the evaluation of candidate genes directly or indirectly involved in the folate metabolic pathway, including *MTHFR*, methionine synthase (*MTR*), betaine–homocysteine methyltransferase (*BHMT*), and serine hydroxymethyltransferase (*SHMT*) to name a few (33, 35, 53). The most compelling observations have been made for the *MTHFR* C677T variant, for which *TT* homozygosity has been significantly associated with NTDs (54). While the cause of folate-dependent NTDs remains unknown, the *TT* genotype results in a thermolabile enzyme and reduced folate metabolism, which may reduce cell proliferation or cellular methylation reactions during embryonic development. Other folate metabolic gene variants including methylenetetrahydrofolate dehydrogenase 1 (*MTHFD1*), dihydrofolate reductase (*DHFR*), methionine synthase reductase (*MTRR*), and the transcobalamin II receptor (*TCblR*) have been associated with NTDs using candidate gene/SNP analysis (55–57).

Candidate gene studies and GWAS to identify SNPs associated with NTD risk have yielded variable results. Three prominent studies examined possible associations between 118 folate pathway-linked SNPs and NTDs. Boyles et al. (35) found a weak association between betaine and homocysteine methyltransferase (*BHMT)*, which also remethylates homocysteine using betaine rather than folate as the methyl donor, and NTD risk when mothers were receiving pre-conceptional folate supplements or when combined with the *MTHFR* (*C677T*) *TT* homozygosity. *BHMT*’s role in NTD pathogenesis is poorly understood, but may be related to altered cellular methylation potential and gene expression. Shaw et al. (33) performed a candidate gene screen of these 118 SNPs in a Californian population, and found a modest association between spina bifida (NTD) risk and SNPs in seven genes related to folate metabolism [*BHMT*, *MTHFD1*, *MTHFD2*, *MTHFR*, *MTRR*, cystathionine-β-synthase (*CBS*), and thymidylate synthase (*TYMS*)]. For nearly all of these variants, (rare) allele homozygosity can result in reduced folate metabolism and its endpoints. An additional analysis of these pathways using haplotype and tagSNP screening approaches identified 68 SNPs associated with NTD risk in genes from the folate metabolic pathway, *MTHFR* and mitochondrial folate transporter (*MFTC*); DNA methylation, DNA (cytosine-5)-methyltransferase 3A (*DNMT3A*); B_12_ metabolism, Cubilin (*CUBN*) and choline metabolism, phosphatidylethanolamine *N*-methyltransferase (*PEMT*) (34).

In contrast, a number of studies have found no association with some of these same gene variants. For example, a SNP in the gene methionine synthase (*MTR A2756G*), in the enzyme required for folate-dependent remethylation of homocysteine, was initially shown to be significantly associated with increased risk for spina bifida in a candidate gene study (58). However, Shaw et al. (33) found no association *MFTC* is a component of the one-carbon transport chain for which it was hypothesized that SNP variants played a role in NTD risk by reducing folate metabolism and DNA methylation. Pangilinan et al. (34) used a tagSNP approach to assess *MFTC*’s role in NTD development and found a minor, but non-significant link with *MFTC* (*R117H*). These confounding observations and lack of a solid answer in these studies may be founded in gene–environment interactions and their sometimes subtle effect on phenotype. For example, none of these studies examined the modifying effect of nutrient intake or status on the relationship between genotypes of interest and NTD risk. The addition of dietary folate and folate status data may resolve discrepancies between studies and improve the confidence in results by providing accountability for a potentially significant environmental factor.

## Cancer

Low folate status has been associated with increased risk for numerous cancers including colon and breast cancer. This association has raised questions including: “Does compromised DNA repair lead to increased cancer risk?” “Could high dietary folate increase the rate of tumor growth?” and “What role does folate consumption or status play in individuals with low-frequency cancer pre-disposition genotypes?” Much like the studies looking at NTDs and folate-related genes, the relationship between cancer, nutrient intake, and gene variants within enzymes involved in folate and B-vitamin metabolism have relied heavily on both candidate gene studies and GWAS (Tables 3 and 4).

**Table 3:** **Major findings observed in association studies examining folate and vitamin B_12_ metabolic pathway genes, using candidate gene studies, major diseases, and dietary folate or B_12_ dietary intake, including sample size, significance (as *p* or 95% confidence interval), and risk measurement (Odds ratio, OR)**.

Topic	Research Study	Findings	Sample Size	Significance	OR
NTD	Doolin et al. (58)	*MTR* (A2756G) NTD association	Total: 209	95% CI: 0.92–5.06	2.16^a^
	Boyles et al. (35)	Weak association between *BHMT* variants (rs3733890, rs558133) in folate rich environments and NTD risk.	Total: 304	*p*: 0.027, *p*: 0.036	
	Parle-McDermott et al. (55)	*DHFR* association with decreased NTD risk.	Cases: 283, controls: 256	95% CI: 0.39–0.89	0.59
	Parle-McDermott et al. (56)	*MTHFD1* (rs3832406) association with increased NTD risk.	Triad n: 439	*p*: 0.002	
	Shaw et al. (33)	Modest association between NTD risk and SNPs in *BHMT*, *CBS*, *MTHFD1*, *MTHFD2*, *MTHFR*, *MTRR*, and *TYMS*.	Cases: 259, controls: 359	*p*: 0.009–0.02	
	Pangilinan et al. (57)	*MTHFD1* R653Q (rs2236225) and *MFTC* (rs17803441) significantly associated with increased NTD risk.	Cases: 301, controls: 341	*p*: 0.0023, *p*: 0.0003	
	Pangilinan et al. (34)	68 SNPs associated with NTD risk including SNPs *MFTC (*rs17803441)*, CDKN2A* (rs3218009)*, ADA* (rs2299686).	Cases: 301, controls: 341	95% CI: 1.23–2.08, *p*: 0.0098, *p*: 0.0004, *p*: 0.0010	1.61
Cancer	Flores et al. (59)	*CBS* (rs2850146) and *MTRR* (rs3776467) SNPs may, in combination with high folic acid levels, protect against lung cancer.	Total: 907	95% CI: 1.98–12.2, *p*: 0.0006	4.9
	Liu et al. (60)	*DNMT 1331V* and *MTRR 122M* significantly interacting with dietary intake to modify CRC risk.	Cases: 1609, controls: 1974	*p*: 0.04, *p*: 0.02	
	Pabalan et al. (61)	No significant association between *SHMT1* (rs1979277) or folate levels and CRC risk.	Cases: 5043, controls: 6311	*p*: 0.47–0.77	

*^a^*r*-Value used in place of OR*.

**Table 4:** **Major findings observed in association studies examining folate and vitamin B_12_ metabolic pathway genes, using GWAS, major diseases and dietary folate and B_12_ dietary intake, including sample size, significance (as *p* or 95% confidence interval), and risk measurement (Odds ratio, OR)**.

Topic	Research Study	Findings	Sample Size	Significance	OR
NTD	Grarup et al. (62)	Whole genome and exome sequencing associated six novel loci with serum B_12_ (*CD320, TCN2, ABCD4, MMAA, MMACHC*) or folate levels (*FOLR3*) with cardiovascular diseases, cancers, and neurodegenerative disorders	Folate: 37341, B_12_: 45576	*p*: 7.11 × 10^−100^ – 0.0018	
Cancer	Vollset et al. (36)	No correlations between gastric cancer and plasma folate, total homocysteine and serum B12. Slight increase in gastric cancer risk with *MTHFR* (rs1801133)	Cases: 245, controls: 631	*p*: 0.04	1.47
	Han et al. (39)	SNPs in *ADA* (rs244072), *CDO1* (rs34869) and *FOLR1* (rs10501409) associated with advanced colorectal adenoma in individuals with extremely low folate intake	Cases: 1331, controls: 1501	*p*: 0.001, *p*: 0.007, *p*: 0.008	
	Tanaka et al. (64)	Significant associations between plasma B_6_ and *ALPL* (rs4654748), serum B12 and *FUT* (rs602662), and homocysteine and *MTHFR* (rs1801133)	*n* = 2930	*p*: 8.30 × 10^−18^, *p*: 2.83 × 10^−20^, *p*: 4.36 × 10^−13^	
	Hazra et al. (63)	Meta-analysis of 3 GWAS confirmed or identified strong associations between B_12_ and *FUT2* (rs602662, rs492602) and between plasma homocysteine and *MTHFR* (rs1081133)	Total: 4763	*p*: 1.83 × 10^−15^, *p*: 1.30 × 10^−14^, *p*: 1.27 × 10^−8^	

Recent studies have examined the association between folate status or consumption, which can be analyzed categorically or continuously, and a limited suite of SNPs in folate-related genes associated with lung, esophageal, stomach, colorectal, and kidney cancers. Examples of key genes that have been studied include *MTHFR, MTHFD, MTR, MTRR*, folate receptor 1 (*FOLR1*), and fucosyltransferase 2 (*FUT2*; a gene associated with vitamin B_12_ status; Tables 2 and 3). The prototype gene studied in relationship to cancer is the *MTHFR* gene, and in particular the *C677T* SNP. The gene product of *MTHFR* plays a key role in folate metabolism, as it produces 5-methylTHF, which is used by MTR for the production of THF and methionine. The *MTHFR C677T* variant results in decreased levels of serum RBC folate and elevated plasma homocysteine. Folic acid supplementation can normalize folate status and enzyme activity, but absolute folate status is lower than in those individuals with the wildtype enzyme.

Since the implementation of folic acid fortification in the United States and Canada (1996 and 1997, respectively), a slowdown in the rate of colorectal cancer (CRC) decline has been suggested to be due to high folic acid intake (65). This has raised many questions regarding the role of folate in CRC risk. Although many studies indicate that high folate status is associated with reduced CRC risk, a handful has suggested that high levels may increase CRC risk in persons with pre-cancerous legions. Liu et al. (60) examined how interactions between folate-related SNP variants and dietary intake (including folate) influence CRC risk. Individuals with *DNMT 1331V* and low folate intake had decreased CRC risk, while higher folate intake increased CRC risk, due in part to enhanced abnormal methylation. The opposite was observed for females with *MTRR* (*122M*) homozygosity, a potential modifier of methionine metabolism. Han et al. (39) suggest that significant pathway- and gene-level associations (*ADA* and cysteine dioxygenase, *CDO1*) exist between one-carbon metabolism genes and advanced colorectal adenoma in individuals with extremely low folate intake. These findings could point to an association between CRC risk and altered homocysteine metabolism. Alternatively, these markers may be in linkage-disequilibrium with unsampled, but relevant genes. A meta-analysis of case–control studies of the *SHMT1 C1420T* SNP found no significant association between gene variants or folate levels and CRC risk (61). The confounding outcomes suggest that further study of the gene–gene interactions are required to resolve the factors leading to pre-disposition and to identify the role of genotypic interactions on phenotypic expression. Using a larger sample-set and/or high-throughput methods may facilitate the study of these interactions at very high resolutions.

Folate-gene association studies have identified more than 100 folate pathway SNPs related to digestive system cancers. In European populations, Vollset et al. (36) found a significant association between the *MTHFR* (*1298* A > C) SNP and increased risk of gastric cancer, potentially due to significantly altered concentrations of circulating folate and homocysteine. Using candidate gene analysis, Zhang et al. (66) indicated that *MTR* (*Ex26_20* A > G) and *MTRR* (*Ex5123* C > T) SNPs are associated with a borderline increased risk of stomach cancer in the Polish population. These polymorphisms may cause increased risk by altering homocysteine to methionine conversions, subsequently increasing homocysteine levels. No significant interactions were observed between dietary folate intake and cancer risk. Though, the findings from both studies indicate that at least three SNP markers are associated with digestive system cancers, their small sample size and regional location may lead to population stratification artifacts, restricting how the results can be extrapolated.

Ibiebele et al. (37) found that nutrient intake played a more important role than gene variants in esophageal cancer risk. Adequate folate intake reduced overall cancer risk, but supplemental folic acid and vitamin B_12_ was significantly associated with increased Barrett’s esophagus and esophageal adenocarcinomas (37). No significant associations were observed between SNPs from the *MTHFR*, *MTRR*, and *MTR* genes and cancer risk, regardless of dietary intake, suggesting that diet may be the critical factor in increasing the risks of these cancers.

Other studies have attempted to link folate metabolic pathway genes with non-digestive tract cancers. Hu et al. (67) performed a meta-analysis and determined that the *MTRR A66G* SNP was not associated with breast cancer risk. A candidate gene study of the *MTHFR* SNPs *C677T* and *A1298C* showed no significant association with increased ovarian cancer risk in a case–control study of American women (38). Flores et al. (59) determined, using a candidate gene SNP study, that SNPs in *CBS* (*8283 G*  > *C*) and *MTRR* (*7068 A*  > *G*) have sex-specific associations with aberrant methylation in the lung epithelium of smokers that could be mediated by their effects on one-carbon metabolism and transsulfuration. It was proposed that in rare cases these variations could act, in combination with high folic acid levels, to protect against lung cancer (68). All of these findings highlight the complex interaction of dietary folate, vitamin B_12_, and homocysteine with genotypic variation.

## Cardiovascular Disease

The link between low intake and status of folate, B-vitamins, high circulating homocysteine, and cardiovascular diseases, including coronary heart disease and stroke has long been a topic of interest (Table 4). The early findings of non-genomic studies suggested that high homocysteine levels in the blood (hyperhomocysteinemia) were weakly correlated to increased risk of cardiovascular disease (69). This has led to the hypothesis that folic acid and B-vitamin supplementation could reduce the risk of cardiovascular disease by lowering homocysteine concentrations. Building on these observations, various candidate gene and GWAS’s were conducted to determine the genetic underpinnings of folate-related cardiovascular disease. The focus of many of these studies was the relationship between genotype and B-vitamin status, homocysteine levels, and cardiovascular disease. Early candidate gene studies found moderate risk associations between high homocysteine and *MTHFR C667T* homozygosity, which would ultimately cause an additional increase in homocysteine, and potentially contribute to increased risk for atherosclerosis and blood clot formation. (70). Tanaka et al. (64) found associations between plasma B_6_ level and *ALPL* (rs4654748 C > T: a component in B_6_ catabolism), plasma B_12_ levels and *FUT* (rs602662 A > G: reduces B_12_ absorption), and homocysteine levels and *MTHFR C667T*. This highlighted potential connections between these metabolic pathways. However, two meta-analyses found no significant correlations between homocysteine, the *MTHFR* C677T SNP, and cardiovascular risks (69, 71). The association between *ALPL* and *MTHFR* SNPs and homocysteine, combined with homocysteine’s role in CVD risk, suggest that these markers may be suitable predictors of plasma homocysteine and also potentially CVD. Expanding the size of study populations combined with new analytical approaches, including continuous trait associations, may improve our ability to identify SNPs that are suitable biomarkers for cardiovascular disease.

## Can SNPs Associated with Folate and B-Vitamin Status and Their Associated Diseases Predict Folate Status?

Our on-going work aims to tease apart the contributions of diet and genetics on folate status and associated disease risk. Only one past study (Table 3) has examined these interactions with regards to folate using a single-marker. The *MTHFR* C677T SNP was shown to reduce folate status and is associated with NTDs and cancer, the risk of which can be mitigated by increased folate intake. We are performing a moderately high-throughput analysis to elucidate similar relationships between a panel of ~120 folate-related SNPs and folate status and their modification by dietary folate intake. The predictive power of key SNPs identified from previous candidate gene and GWAS analyses will allow us to estimate proportions of the population that may be most impacted by folic acid intake from food fortification and supplement use. This could include identifying groups that derive particular benefit from the consumption of additional folic acid as well as those individuals that may be at risk for over-exposure. Consideration of folate intake may also reduce the ambiguity of results from previous studies that did not control for this obvious confounding variable.

We have designed a two step analysis to address this issue using DNA samples from the Canadian Health Measure Survey, cycle 1 with a samples size of approximately 3200 adults aged 20 years and older. Information on the participants’ folate and vitamin B12 dietary intake and supplementation was collected, and red blood cell folate, serum vitamin B12, and plasma total homocysteine were measured. The first step of our analysis will examine the association between candidate SNPs, which were previously associated with nutrient status or disease, and the phenotypic variables red blood cell folate, plasma vitamin B_12_, and plasma total homocysteine using single-marker and multi-marker (haplotype and gene–gene interaction) approaches. The multi-marker analysis may reveal multi-gene interactions that influence an individual’s nutrient status. The analysis will be expanded in the second step with the addition of the environmental variables, including dietary, and supplement intake of the nutrients, to identify SNPs that modify the expected relationship between nutrient intake and status. Ultimately, we hope to refine our understanding of the relationship between these SNPs and nutrient status by controlling for nutrient intake, as well as identifying SNPs that may result in hypo- or hyper-responsiveness to nutrient intake (e.g., SNPs that result in lower or higher than expected status).

The data from studies such as we are performing will help to inform, update, and refine policies, regulations, and recommendations to maximize the benefits of folic acid while minimizing the risks of over-exposure to genetically susceptible sub-populations. The general approach, we are proposing can be applied to other nutrients and environmental factors to begin to understand the implications of food policies and regulations for individuals in the general population. The ultimate goal of public health policy is to improve the quality of life and health for the majority of a given population. Improved understanding of the relationship between nutrient intake, genetic variation, and nutrient status or disease may therefore allow for the refinement of nutrition policies.

## Conflict of Interest Statement

The authors declare that the research was conducted in the absence of any commercial or financial relationships that could be construed as a potential conflict of interest.
